# Influence of cytokines, circulating markers and growth factors on liver regeneration and post-hepatectomy liver failure: a systematic review and meta-analysis

**DOI:** 10.1038/s41598-021-92888-4

**Published:** 2021-07-02

**Authors:** Anastasia Murtha-Lemekhova, Juri Fuchs, Omid Ghamarnejad, Mohammedsadegh Nikdad, Pascal Probst, Katrin Hoffmann

**Affiliations:** 1 Department of General, Visceral, and Transplantation Surgery, Ruprecht Karl University, Im Neuenheimer Feld 110, 69120 Heidelberg, Germany; 2 Study Center of the German Surgical Society (SDGC), Heidelberg University Hospital, Heidelberg, Germany

**Keywords:** Biomarkers, Diseases, Gastroenterology, Health care, Medical research, Pathogenesis

## Abstract

The pathophysiology of post-hepatectomy liver failure is not entirely understood but is rooted in the disruption of normal hepatocyte regeneration and homeostasis. Current investigations of post-hepatectomy liver failure and regeneration are focused on evaluation of circulating hepatic function parameters (transaminases, cholestasis, and coagulation parameters), volumetry and hepatic hemodynamics. However, identification of biochemical factors associated with regeneration and post hepatectomy liver failure is crucial for understanding the pathophysiology and identification of patients at risk. The objective of the present systematic review was to identify circulating factors associated with liver regeneration and post hepatectomy liver failure in patients undergoing hepatectomy. The quantitative analysis was intended if studies provided sufficient data. Electronic databases (MEDLINE via PubMed, Web of Knowledge, Cochrane Library and WHO International Clinical Trials Registry Platform) were searched for publications on cell signaling factors in liver regeneration and post-hepatectomy liver failure following liver resection in clinical setting. No date restriction was given. No language restriction was used. Studies were assessed using MINORS. This study was registered at PROSPERO (CRD42020165384) prior to data extraction. In total 1953 publications were evaluated for titles and abstracts after exclusion of duplicates. Full texts of 167 studies were further evaluated for inclusion. 26 articles were included in the review and 6 publications were included in the meta-analyses. High levels of serum hyaluronic acid even preoperatively are associated with PHLF but especially increased levels early after resection are predictive of PHLF with high sensitivity and specificity. Postoperative elevation of HA to levels between 100 and 500 ng/ml is increased the risk for PHLF ([OR] = 246.28, 95% [CI]: 11.82 to 5131.83; *p* = 0.0004) Inteleukin-6 levels show contradicting result in association with organ dysfunction. HGF positively correlates with liver regeneration. Overall, due to heterogeneity, scarcity, observational study design and largely retrospective analysis, the certainty of evidence, assessed with GRADE, is very low. High levels of serum hyaluronic acid show a strong association with PHLF and increased levels after resection are predictive of PHLF with high sensitivity and specificity, even on POD1. Interleukin-6 levels need to be studied further due to contradictive results in association with organ dysfunction. For HGF, no quantitative analysis could be made. Yet, most studies find positive correlation between high HGF levels and regeneration. Prospective studies investigating HGF and other growth factors, hyaluronic acid and interleukins 1 and 6 in correlation with liver regeneration measured sequentially through e.g. volumetry, and liver function parameters, preferably expanding the analysis to include dynamic liver function tests, are needed to sufficiently illustrate the connection between biomolecule levels and clinical outcomes.

## Introduction

The dynamics behind the liver’s unprecedented regeneration capacity are only partially understood. Simultaneously, liver resection is the treatment of choice for primary and secondary hepatic malignancies and numerous benign lesions^1–3^. In a healthy liver up to 80% of the parenchyma may be removed without detriment to the individual. Experienced hepatobiliary surgeons use this quality perform extensive operations such as hemihepatectomies or trisectionectomies with curative intent^4,5^.

Most studies investigating liver regeneration and post-hepatectomy liver failure focus on identification of patient-related, clinical and intraoperative risk factors^6^. A factor exhaustively explored in association with PHLF is the future liver remnant volume^4^. Once the liver has been damaged due to chemotherapy, metabolic disease or various noxas, it develops steatosis, fibrosis or cirrhosis—parenchyma marked by reduced regeneration capacity^7^. In these cases, at least 30–40% of total functional liver volume must be preserved to prevent small-for-size syndrome (SFSS) and consequent post-hepatectomy liver failure^8^. Recent topic to regain popularity is the hepatic inflow in major resections and numerous models show correlation between high portal influx and PHLF^9^. Relative overperfusion and increased portal venous pressure have been sufficiently shown as factors reducing regenerative capacity^10^. Identification of all these factors has made extensive and safer hepatobiliary surgery feasible, however PHLF remains one of the most threatening conditions and occurs in up to 30% of cases^11^.

Only few studies investigating the dynamics of liver regeneration focus on cellular and biochemical processes. Animal studies show that liver regeneration is initiated immediately after resection^12^. Hepatocytes, otherwise dormant cells in the G0 phase of the cell cycle, are not terminally differentiated, as they preserve the ability to divide, and unfold a high proliferative capacity after parenchymal injury^13^. After hepatic resection, diploid hepatocytes proliferate, leading to a rapid increase in liver volume within ten days^12^.

The liver regeneration process can be generally separated into three stages:

1. The priming phase commences immediately after liver injury, is driven by an influx of gut-derived lipopolysaccharides by inflammatory cells and sensitizes hepatocytes to circulating growth factors^14^. Within a few minutes after liver resection a cytokine burst is triggered predominated by tumor necrosis factor-α (TNF-α) and interleukin-6 (IL-6). Over 100 early response transcription factors are triggered and this induces hepatocytes to re-enter G1 stage of the cell cycle^15^. This phase is characterized also by the increased portal venous flow and sheer stress associated changes in the liver sinusoidal endothelial cells, such as WNT protein production^16^.
2. In the progression phase hepatocyte growth factor (HGF), transforming growth factor α (TGF-α), epidermal growth factor (EGF), heparin-binding EGF-like growth factor (HB-EGF) activate delayed gene transcription factors and stimulate DNA synthesis and cell proliferation^13,15^. The progression phase is also called the proliferation phase due to abundance of hepatocytes undergoing mitosis.
3. Once regeneration progressed sufficiently, the termination phase commences, induced foremost by TGF-β, as well as integrin-linked kinase^12,17^. This stage of regeneration is very poorly understood. However, evidence suggests that TGF-β1 secreted by spleen plays an important role in the process. TGF-β1 downregulates HGF and splenectomy leads to an increased proliferation after hepatectomy^18^.

Simultaneously, splenomegaly and thrombocytopenia have been associated with post-hepatectomy liver failure^19^ highlighting the importance of platelets and platelet-derived biomolecules in liver regeneration^20^. Most findings are extracted from animal models and cell cultures and are restricted in their translation to clinical practice^12,21,22^. Mathematical models that are increasingly used in research are also largely based on animal models^23,24^.

Few studies focus on identification of serological markers for liver regeneration or post-hepatectomy liver failure. The aim of this systematic review is to identify potential clinically predictive markers for adequate liver regeneration or PHLF in patients undergoing hepatectomy.

## Methods

This systematic review and meta-analysis is reported in accordance with the PRISMA guidelines^25^. The study was registered with PROSPERO (CRD42020165384)^26^.

### Literature search

A systematic literature search was conducted in accordance with the recent recommendations of the Cochrane Collaboration^27^. The searches aimed to identify all published and unpublished studies reporting influence of cytokines, growth factors and circulating markers on liver regeneration or PHLF in patients undergoing hepatectomies^28^. The search strategy was chosen based on a recent review on circulating factors in liver regeneration that largely analyzed animal models^14^. The searches of the of the electronic databases MEDLINE via PubMed, Web of Knowledge, Cochrane Library and WHO International Clinical Trials Registry Platform) were performed. The search strategy contained key search terms “hepatectomy”, “post-hepatectomy liver failure”, and their synonyms, as well as cell signaling molecules “hepatocyte growth factor”, “tumor necrosis factor alpha”, “interleukin 6”, “epidermal growth factor”, “insulin-like growth factor”, “vascular endothelial growth factor”, “fibroblast growth factors”, “angiopoietin”, and “platelet-derived growth factor”. The search strategy for MEDLINE (via PubMed) is shown in Appendix 1. Similar search strategy was used for other platforms. Additionally, hand-searches were performed through the reference lists of review articles and reports of clinical trials to identify further relevant studies. The last search was performed on July 23, 2020. No publication year restriction was placed on the search. No language restriction was made.

### Study selection

No restriction was placed on method of the study. Studies evaluating cell-signaling molecules after liver resection during liver regeneration phase or in relation to post-hepatectomy liver failure in humans were eligible for inclusion. A scarcity of studies examining the endpoints was anticipated, thus no study design was excluded. All other publications such as animal studies, abstracts from meetings, comments, correspondence, and editorials were excluded. Publications for which the full text could not be procured were also excluded. Prior to final exclusion, authors were contacted directly for the full text if it was not available through established access. The screening of titles, abstracts and full texts was carried out by two independent reviewers. All disagreements were resolved by consensus and consultation with a third reviewer.

### Data extraction

Data was extracted from studies that met the final inclusion criteria by two reviewers using a standardized form. Following data was extracted: title of the publication, year, author, country, journal, study design, number of study groups, total number of patients, patient characteristics, factors, indications for hepatectomy, if and type of underlying liver disease was present, type of hepatectomy and outcomes. The reviewers also noted the sources of funding for the studies included in this review. The outcomes of interest were post-hepatectomy liver failure, defined as excessive hyperbilirubinemia (> 10 mg/dl) or according to ISGLS guidelines^29^ and liver regeneration assessed by liver volumetry or clinical parameters.

### Statistical analysis

Meta-analyses were performed using Review Manager 5.3. Forest plots were used to present effect estimates. For all outcomes, a random-effect model was applied due to heterogenic methodological and clinical framework of the studies. Statistical heterogeneity among the effect estimates of the included trials was evaluated using the I2 statistic. An I2 value of less than 25% was considered to indicate low heterogeneity and over 75% to indicate high heterogeneity. Odds ratios and 95% confidence intervals were pooled for dichotomous outcomes using the Mantel–Haenszel method. Continuous outcomes were pooled as weighted mean difference (MD) with 95% confidence interval using the inverse-variance method.

## Results

**Figure 1:**
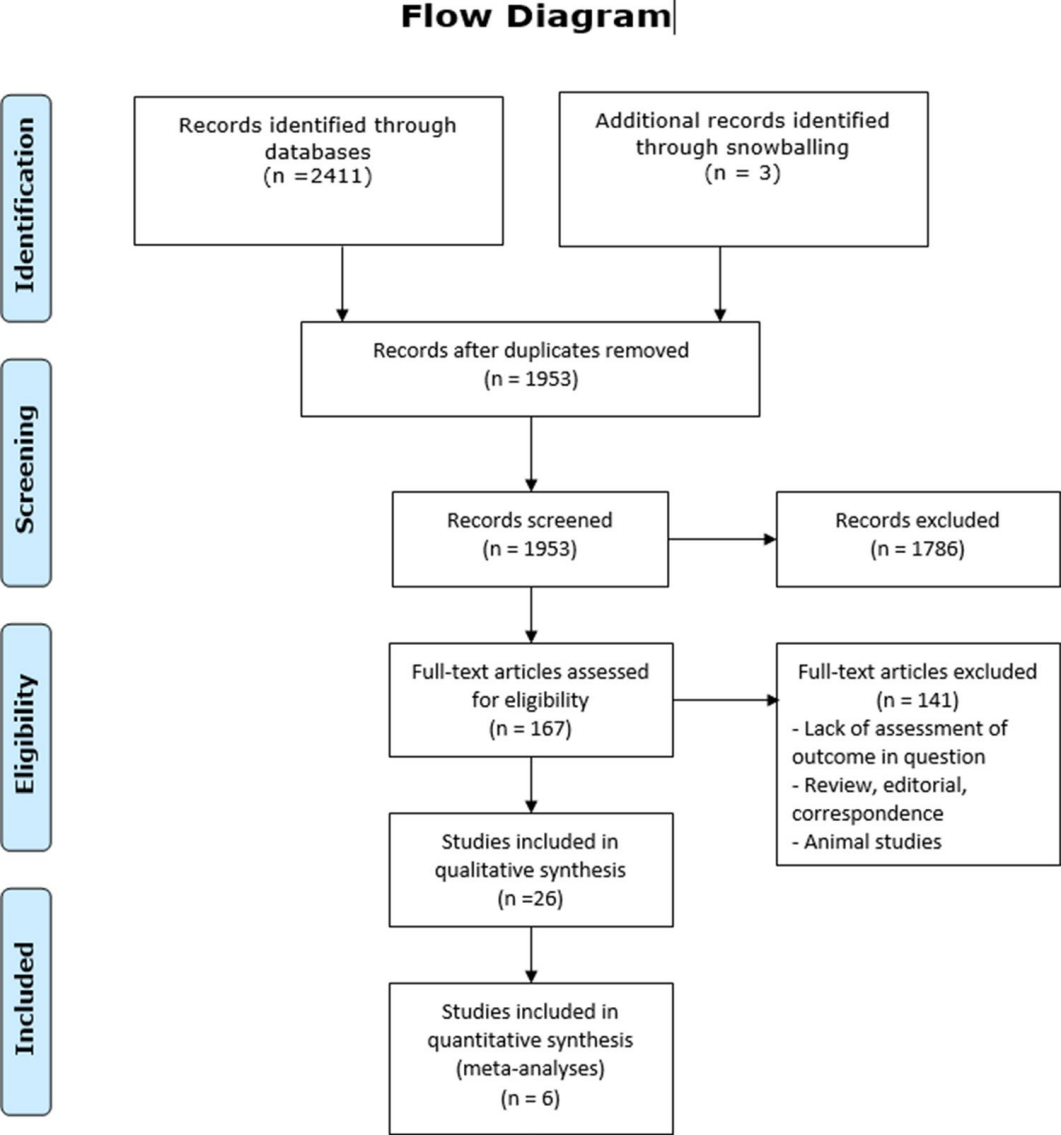
Flow chart of the study selection process.

A total of 2414 records were identified through searches. 458 publications were excluded as duplicates. 167 articles were assessed by full-text review. 26 studies were included in qualitative synthesis and 6 studies were further included in the quantitative analyses. Figure 1 provides an overview of the study selection process for the systematic review (Fig. 1). Based on the results of the full-text analysis, following markers were evaluated: hepatocyte growth factor (HGF), vascular endothelial growth factor VEGF, interleukin-1 (IL-1), interleukin-6 (IL-6) and serum hyaluronate (HA). Quantitative analysis was possible for IL-6 and HA.

### Critical appraisal of included studies

The assessment of quality of non-randomized studies was performed using MINORS for cohort studies included in the quantitative analysis^30^ (Table 1) and included assessment of stated aims, inclusion of consecutive patients, prospective collection of data, appropriate endpoints, bias, appropriate follow-up period with loss of less than 5% and if prospective calculation of the study size was utilized. Quality assessment was not performed for studies included in the qualitative analysis due to heterogeneity of outcome measurements and scarcity of comparable outcomes. Quantitative and qualitative results were merged for each factor studied.

**Table 1:** MINORS.

	A clearly stated aim	Inclusion of consecutive patients	Prospective collection of data	Endpoints appropriate to the aim of the study	Unbiased assessment of the study endpoint	Follow-up period appropriate to the aim of the study	Loss to follow-up less than 5%	Prospective calculation of the study size	Total
Das et al. (2001)^35^	2	2	0	2	0	2	0	0	8
Kimura et al. (2006)^39^	2	2	0	2	0	2	0	0	8
Maeda et al. (1999)^41^	2	0	0	2	0	2	0	0	6
Mizuguchi et al. (2004)^54^	2	2	1	2	0	2	0	0	9
Yachida et al. (2000)^55^	2	1	1	2	0	2	0	0	8
Yachida et al. (2009)^56^	2	0	1	2	0	2	0	0	7

### IL-1

Two observational single cohort studies measured IL-1 in patients undergoing hepatectomy^31,32^. Both studies noted an increase of concentration shortly after resection despite one study only investigating patients undergoing hepatectomy with hepatic vascular exclusion (HVE)^32^. Clavien et al. found statistically significantly higher IL-1 levels in patients suffering from cirrhosis compared to non-cirrhotic patients^31^. In both studies, IL-1 levels returned to baseline within 24 h after resection and no correlation was reported to postoperative outcomes.

### IL-6

Thirteen single cohort observational studies, described serum IL-6 concentration in association with liver regeneration or PHLF^31–43^. Seven studies described peak concentration on first postoperative day after hepatectomy, with mean values ranging from 55.5 to 1027 pg/ml^34,38–43^. One study described the IL-6 peak concentration between day of operation and first postoperative day^37^ and two studies reported the peak on the day of the operation during the early post-hepatectomy phase^32,36^.

**Figure 2:**
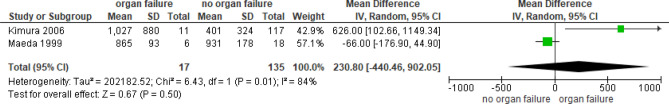
Forrest plot of studies examining IL-6 levels and organ failure.

Both, Cata et al. and Das et al. describe significantly higher levels of IL-6 in patients with complications compared to those without^34,35^. Clavien et al. reported a statistically significant negative correlation between interleukin-6 at the end and 5 min after hepatectomy and number of postoperative complications, however interleukin was measured in the portal vein^31^. Two studies investigated association between IL-6 levels and organ dysfunction^39,41^. Kimura et al. described significant elevation in patients with organ dysfunction while Maeda et al. showed significant elevation in patients without dysfunction, although the second study investigated association with liver function alone. In the meta-analysis, there was no significant association between IL-6 concentration and organ dysfunction (*p* = 0.50) (Fig. 2).

Three studies showed a positive correlation between liver volume/mass resected and interleukin-6 levels^33,36,38^. One study showed a positive association between major resections and interleukin-6 levels^33^, while another showed no significance in that respect^37^.

### HGF

Thirteen single cohort observational studies were identified that analyzed HGF levels in association with liver regeneration^36,40,42–52^. One study additionally investigated the association of serum HGF and PHLF and described higher serum HGF levels in patients without liver failure^49^.

Several studies described a significant increase of HGF post resection with a peak by POD1^36,40,44,47–50^. One study noted a peak on second postoperative day (POD2) after hepatectomies for metastatic disease versus hepatomas and showed higher serum HGF values in hepatomas generally^50^. Both findings contradict results later described by Dluzniewska et al.^44^. Another study described HGF peak on POD1 for cirrhotic patients, while patients without cirrhosis peaked on POD2 and had overall lower values compared to patients with cirrhosis that underwent partial hepatectomy^52^. The peak of serum HGF varied greatly among studies with values ranging from ca. 180 to ca. 2250 pg/ml. However, inclusion criteria in these studies were heterogeneous, ranging from minor to major liver resections for various benign and primary, as well as metastatic liver tumors.

Three studies examined a correlation between extent of resection and HGF. While one study reported an inverse correlation^48^, two others did not find any association between extent of resection and serum HGF^47,52^. However, all studies used different measurements for extent of resection: ratio of remnant liver volume per body weight on POD0, percent resected liver volume (%RLV) and number of segments resected.

Four studies investigated the correlation between remnant liver growth and serum HGF^43,48,50,52^. However, all studies used different measures of liver growth: ratio of liver volume on POD14 to liver volume on POD0, growth of functioning liver remnant at POD1 after second stage of ALPPS (associating liver partition and portal vein ligation for staged hepatectomy) and the percentage of increase in the remnant liver volume after 28 days or 6 months after surgery. One of these studies investigated only patients undergoing ALPPS procedure^43^. While three studies showed a correlation between remnant liver growth and serum HGF^43,48,50^, one did not^52^. Due to different statistical analyses used to calculate correlation and a lack of raw data, no meta-analysis was performed for HGF relationship to regeneration surrogate parameter.

### VEGF

Five studies, four prospective and one unspecified observational cohort studies, could be identified exploring VEGF in relationship with PHLF and regeneration^33,45,46,51,53^. Significant elevation of VEGF was described on postoperative days one through five. One study suggested VEGF is significantly elevated when no postoperative liver dysfunction is present^53^.

One study described a positive correlation between VEGF concentration and resected volume^33^. Similarly, Effimova et al. showed higher VEGF concentrations in patients undergoing right hepatectomy for liver donation compared to patients after liver resection for HCC, although the extent of resection in HCC patients ranged from wedge resection to an extended hemihepatectomy^45^.

### HA

Four studies, three retrospective and one unspecified observational cohort studies, investigated serum hyaluronic acid in relation to PHLF and liver function^35,54–56^. Three studies have described a positive correlation between preoperative serum HA and ICGR15 to various degrees^35,54,55^. Only one study examined correlation between serum HA and several liver function parameters. Mizuguchi et al.^54^ found serum HA to significantly correlate with lectin-cholesterol acyltransferase (LCAT), HGF, serum prealbumin (pre-ALB) and scintigraphy liver assessment parameters: liver uptake of Tc-99 m-GSA (HH15) and the hepatic uptake ratio (LHL15).

**Figure 3:**
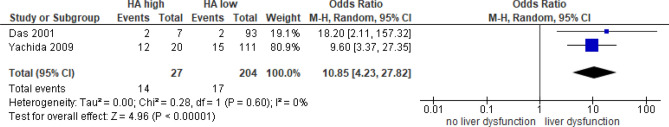
Forrest plot of studies examining preoperative serum HA levels and liver dysfunction.

Two studies with total of 204 patients analyzed the association of preoperative hyaluronic acid with the development of PHLF^35,56^. Pooled analysis showed that high preoperative HA (above the cut-off defined by Yachida et al. at 180 ng/ml and by Das et al. at 200 ng/ml) was associated with a higher risk for postoperative liver dysfunction ([OR] = 10.85, 95% [CI]: 4.23 to 27.82, I2 = 0%) (Fig. 3).

**Figure 4:**
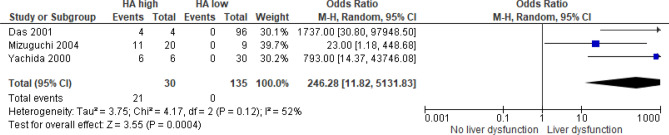
Forrest plot of studies examining postoperative serum HA levels and liver dysfunction.

Three studies, including a total of 135 patients, analyzed the association of postoperative serum HA with the occurrence of PHLF^35,54,55^. Pooled analysis suggests that postoperative elevation of HA was indicative of PHLF with no reports of postoperative liver dysfunction in patients with postoperative serum HA levels under the cut-off values ([OR] = 246.28, 95% [CI]: 11.82 to 5131.83, I2 = 52%, *p* = 0.0004) (Fig. 4). The cut-off values for high postoperative HA were placed by the authors between 100 and 500 ng/ml. Two studies^35,55^ measured serum HA on first, one^54^ only on the seventh postoperative day.

### Certainty of evidence (GRADE)

A rating on quality of the evidence for every outcome was made with the Grading of Recommendations Assessment, Development and Evaluation (GRADE) approach^57^ (Table 2). Overall, the quality of evidence was very low in all outcomes considered. This is due to the study design (observational, mostly retrospective or unspecified) and due to suboptimal information within the publications.

**Table 2:** GRADE summary of findings.

Outcome	Effect (95% CI)	Pooled prevalence (CI 95%)	Number of studies	Number of participants	Certainty of evidence (GRADE)
IL-1 as diagnostic factor for PHLF	–	–	2	23	Very low
IL-6 as diagnostic factor for organ dysfunction	MD (95% CI): 230.80 (− 440.46–902.05)	–	2	152	Very low
PreHA as a diagnostic factor for PHLF	Sensitivity: 45.16% (27.32–63.97%) Specificity: 93.50% (89.14–96.49%)	13.42% (9.30–18.50%)	2	231	Very low
PostHA as diagnostic factor for PHLF	Sensitivity: 100% (83.89–100%) Specificity: 93.75% (88.47–97.10%)	12.73% (8.05–18.79%)	3	165	Very low
HGF as diagnostic factor for PHLF	–	–	1	24	Very low
VEGF as diagnostic factor for PHLF	–	–	5	250	Very low

## Discussion

Major resection remains the most perilous procedure within the hepatobiliary surgery spectrum spare for transplantation. Although extensive resections are becoming increasingly safer as clinical research and surgical technique progress, patients still demonstrate high mortality and morbidity rates^58–60^. The most important postoperative complication is the post-hepatectomy liver failure. It occurs due to inadequate remnant liver volume, high post-hepatectomy inflow or damaged parenchyma, all leading to insufficient liver regeneration^11,61^.

There is a multitude of studies investigating liver regeneration, mostly focusing on animal models. Although basic models yield valuable information on the regeneration dynamics, data gathered from a controlled model is hardly inferable for clinical practice. Sporadic studies investigate circulating factors in human subjects undergoing liver surgery. Out of the paucity of studies only few factors could be identified so far that show promising clinical relevance, and investigation of factors identified from animal models can provide further insight into liver regeneration after hepatectomy. VEGF, HGF and IL-6 have been described as promising regeneration predictive markers in mice, with VEGF-A application leading to a significant increase in liver volume and recombinant human HGF treatment simulating proliferation in rats^14^. These findings are parallel to data in human studies. IL-6, interestingly, enabled hepatocyte proliferation and prevented postoperative mortality in mice, yet, in humans, higher levels of IL-6 were associated with organ failure^14,37^. Other factors identified through animal studies are yet to be investigated in humans, e.g. insulin-like growth factor 1 that has been shown to induce regeneration after liver injury^14^.

### IL-1

Interleukin-1 is a member of acute-phase proteins that increases in various conditions of inflammation. The interleukin-1 family of cytokines has mostly been studied in the context of sepsis and autoimmune diseases^62^. IL-1 α precursor is constantly present in healthy hepatocytes and is released in necrosis^63^, a process unavoidable at the resection area after hepatectomy. IL-1 β is not transcribed in a healthy liver tissue^63^. Interestingly, Clavien et al. described higher levels of IL-1 in cirrhotic patients compared to non-cirrhotic^31^, although unknown if α- or β-subtype. Guidi et al. measured IL-1 α but did not find a correlation between cytokine concentration and liver parameters^32^. Both studies investigated the effect of vascular occlusion on patients and cytokine levels with Clavien et al. randomizing for hepatic vascular exclusion and Guidi et al. describing a cohort that underwent portal venous clamping^31,32^.Both studies assessed a small cohort (15 and 16 patients) and differed extensively in the study design (randomization for hepatic vascular occlusion versus prospective observational study) and endpoints (comparison of liver cirrhotic vs non-cirrhotic patients and not differentiated cohort). Overall, the certainty of evidence if very low for the question if IL-1 as a predictor for PHLF.

Especially IL-1 α is an interesting parameter to assess in relation to PHLF and regeneration, since it may correlate to the level of necrosis. For now, interleukin-1, both alpha and beta, remains a poorly studied cytokine in hepatobiliary surgery.

### IL-6

Interleukin-6 is an inflammation-specific cytokine synthesized by fibroblasts, endothelial cells, T-cells, monocytes and macrophages^64^. Liver injury inevitably leads to an inflammation process, especially in the area of the resection. This causes a flood of inflammatory cells and paracrine torrent of inflammatory cytokines, such as TNF-α and IL-1^65^. These cytokines stimulate in turn IL-6 production and release. Moreover, liver resection causes dramatic changes in liver hemodynamics. Due to portal hyperperfusion, gut bacteria derived lipopolysaccharides increasingly stimulate Toll-like receptor-4 on Kupffer cells and lead to additional release of IL-6^66^.

Multiple studies showed IL-6 peaks on POD1 after hepatectomy with significant differences in concentration across these studies. Despite very low certainty of evidence due to heterogeneity of study designs and outcomes reported, since all studies reported a postoperative elevation of IL-6, this should be considered a normal phase of liver regeneration after a surgical assault. Whether IL-6 is significantly elevated in patients who develop organ dysfunction or rather those without remains unclear. Kimura et al. showed significant elevation of IL-6 in patients with organ dysfunction^37^. Maeda et al., on the other hand, analyzed patients with liver failure and showed higher levels of IL-6 in patients without liver dysfunction^41^. The second study had a significantly lower sample size compared to Kimura’s, with only 24 patients versus 128 but had a more specific study design, targeting major resections for primary liver cancers (cholangiocarcinoma and gallbladder carcinoma) and analyzing liver dysfunction rates.

Since IL-6 is an unspecific proinflammatory cytokine it is plausible that certain level of inflammation and therefore IL-6 concentration is beneficial for liver regeneration while excessive levels are potentially harmful for healthy organs. Further study into optimal levels of IL-6 in liver regeneration after hepatectomy would be useful, especially with an emphasis on determining the threshold for damage to other organ systems.

### HGF

Hepatocyte growth factor was initially identified in 1984 as a humoral mitogenic hepatotropic protein^67^. Although original conjecture was that HGF is a major factor in liver regeneration due to high concentrations following liver injury and hepatectomy, HGF targets not only hepatocytes, hepatoblasts and bile duct epithelial cells. HGF, also known as scatter factor and tumor cytotoxic factor is an important ligand in pancreatic β islet cells, gastrointestinal epithelial cells, renal tubular cells, alveolar type II epithelial cells and even in the nervous system (including hippocampal and midbrain dopaminergic neurons)^68^. HGF is produced in the mesenchymal cells and can be found in abundance in the liver matrix^69^. HGF has been extensively studied in murine models^70,71^, however only few studies exist concerning HGFs role in human liver regeneration.

After hepatectomy, HGF level rises rapidly following IL-6 and TNF stimulation and peaks on the first postoperative day (POD1), reaching 0.5–2 ng/ml and stays elevated for 7–14 days after the resection^43,44^. Later peaks have also been described^42,51^. It is unclear if HGF correlates with remnant liver volume. Matsumoto et al. extensively analyzed correlations between HGF levels and liver volume, and reported an inverse correlation between remnant liver volume per body weight on POD0 to serum HGF on POD1^48^. A later study by Krieg, however showed no association between extend of resection—an inverse parameter for remnant liver volume—and HGF^47^. Three studies found a positive correlation between postoperative serum HGF levels and remnant liver growth^43,48,50^. It is plausible that HGF level is not dependent of the extent of liver injury but is rather a marker for regenerative dynamic after liver resection. Only Sparrelid studied perioperative HGF levels in patients undergoing ALPPS, a procedure that supports and utilized liver’s unique ability to regenerate rapidly upon surgical stress^43^.

Interestingly, bile HGF was found to be significantly increased in patients without liver failure and a negative correlation has been shown between bile HGF and serum bilirubin^49^. Possibly, sufficient liver regeneration is dependent on appropriate HGF levels. However, altogether, the certainty of evidence for HGF as a diagnostic marker for PHLF or adequate regeneration is very low.

Studies determining adequate levels of HGF after hepatectomy are necessary to ascertain if a cut-off value is indicative of sufficient regeneration and can be clinically used to assess post-hepatectomy outcomes.

### VEGF

After initial liver regeneration by hypertrophy of hepatocytes, angiogenesis and the sinusoidal web are reestablished, regulated largely by VEGF^14^. VEGF stimulates metalloproteinases and promotes growth and division of endothelial cells, smooth muscle cells and fibroblasts necessary for new blood vessel development^72^.

Unlike HGF, VEGF correlates to resected volume^33,45^. Whether or not VEGF concentration correlates with growth of liver remnant in patients undergoing hepatectomy remains unanswered. Animal studies suggest a positive correlation^73^ but this remains to be shown in a clinical setting. Interestingly, Starlinger et al. analyzed VEGF levels and postoperative liver dysfunction and found high levels in patients with favorable outcome, suggesting VEGF as a predictor of sufficient regeneration, however this is the only identified study so far concerning this outcome^53^.

### HA

Hyaluronic acid is a glycosaminoglycan present in the extracellular matrix of practically every organ^74^. In the liver, hepatic stellate cells synthesize HA which later is degraded by sinusoidal endothelial cells. HA has been established as a serum biomarker for severe hepatic fibrosis in chronic liver disease^75^. Low levels of HA enter the blood stream through the lymphatic system but get cleared quickly by the liver endothelial cells^74^. High levels of serum HA however are indicative of liver damage and fibrosis and HA was even proposed as a serum marker for staging of fibrosis and as a determinant for initiation and selection of hepatitis C treatment^76^.

The results of this review suggest serum HA concentration is predictive for PHLF. Preoperatively increased serum HA may be indicative of an undiagnosed liver damage prior to hepatectomy which explains higher PHLF rates in this group. Postoperatively high serum HA was shown to be predictive for PHLF with high sensitivity (100%) and specificity (93.75%). An important aspect is that serum HA were measured on the first postoperative day in most studies. These findings suggest that HA is a clinically relevant parameter to be used as a diagnostic criterium for PHLF as well as a preoperative assessment of underlying liver disease. A large prospective randomized cohort study is essential to elucidate predictive diagnostic value of preoperative and postoperative HA levels for PHLF. It would also be interesting to examine the correlation between serum HA concentrations and liver regeneration.

Although the results of this systematic review and meta-analysis stress the importance of hyaluronic acid as predictor for PHLF whether taken preoperatively or postoperatively, the certainty of evidence is very low.

## Conclusion

The results of this systematic review and meta-analysis emphasize hyaluronic acid as a predictive marker for PHLF with high sensitivity and specificity, although the certainty of evidence is very low. Hyaluronic acid should be further investigated prospectively in its relevance in the diagnosis of acute liver failure after hepatectomy and as a preoperative factor for eligibility of patients undergoing hepatectomy. Moreover, we showed that HGF correlates with liver volume increase and optimal levels of HGF should be determined for better assessment of patients after hepatic resection. Predictive value of IL-6 remains unclear. An in-depth study into optimal concentration of this cytokine after hepatectomy may improve our understanding of inflammation as a stimulus for regeneration. Study of IL-1 elevation after hepatectomy is also sensible as its relevance in liver surgery remains unclear. Especially elucidation of the relationship with necrosis may lead to some interesting hypotheses. Lastly, VEGF’s role in liver response after hepatectomy continues to elude and studies into levels of VEGF after various hepatectomies may lead to exciting results.

Overall, prospective studies investigating HGF and other growth factors, hyaluronic acid and interleukins 1 and 6 in correlation with liver regeneration, measured sequentially through e.g. volumetry, and liver function parameters, preferably expanding the analysis to include dynamic liver function tests, are needed to sufficiently illustrate the connection between these biomolecule levels and clinical outcomes.

## Supplementary Information

Supplementary Information 1.

Supplementary Information 2.

## Data Availability

All data generated or analysed during this study are included in this published article and its supplementary information files.
