# Supplementary material for: Influence of cytokines, circulating markers and growth factors on liver regeneration and post-hepatectomy liver failure: a systematic review and meta-analysis
Source: Sci Rep. 2021 Jul 2;11:13739. doi: 10.1038/s41598-021-92888-4 (PMC8253792; doi:10.1038/s41598-021-92888-4)
Supplement: Supplementary file 1 — Supplementary Information 1. [file 41598_2021_92888_MOESM1_ESM.docx]

Appendix 1:

Search strategy for MEDLINE (via PubMed)

("hepatectomy"[All Fields] OR "liver resection"[All Fields] OR "hemihepatectomy"[All Fields]) AND ("post-hepatectomy liver failure"[All Fields] OR "Hepatocyte growth factor"[All Fields] OR "Tumour necrosis factor alpha"[All Fields] OR "Interleukin 6"[All Fields] OR "Epidermal growth factor"[All Fields] OR "Insulin-like growth factor"[All Fields] OR "Vascular endothelial growth factor"[All Fields] OR "Fibroblast growth factors"[All Fields] OR "Angiopoietin"[All Fields] OR "Platelet-derived growth factor"[All Fields])
