# Supplementary material for: Influence of cytokines, circulating markers and growth factors on liver regeneration and post-hepatectomy liver failure: a systematic review and meta-analysis
Source: Sci Rep. 2021 Jul 2;11:13739. doi: 10.1038/s41598-021-92888-4 (PMC8253792; doi:10.1038/s41598-021-92888-4)
Supplement: Supplementary file 2 — Supplementary Information 2. [file 41598_2021_92888_MOESM2_ESM.docx]

Supplementary Table 1: Overview of included studies for the systematic review

| **Study, year**  **(reference)** | **Population**  **(cases)** | **Study design** | **Duration (years)** | **Factor(s)** | **Included for Outcome(s)** |
| --- | --- | --- | --- | --- | --- |
| Aryal 2016 (32) | hepatectomy for HCC (37) | Prospective cohort observational study | 2013 – 2014 | VEGF-A, IL-6 | Liver injury / regeneration |
| Cata 2017 (33) | Hepatectomy for various lesions (40) | Prospective cohort observational study | 2013 – 2015 | IL-6 | Liver injury / regeneration |
| Clavien 1996 (30) | Hepatectomy for various lesions (15) | Randomized for vascular occlusion, Not specified | Not specified | IL-1, IL-6 | Liver injury / regeneration |
| Das 2001 (34) | Hepatectomy for various lesions (100) | Not specified | 1994 – 1998 | IL-6, HA | Liver failure / regeneration |
| de Jong 2001 (35) | Hepatectomy for various lesions (14) | Prospective cohort observational study | 1996 – 1998 | IL-6, HGF | Liver injury / regeneration |
| Dluzniewska 2002 (43) | Hepatectomy for metastasis of metachronous colorectal carcinoma (25) | Not specified | Not specified | HGF | Liver injury / regeneration |
| Effimova 2004 (44) | Hepatectomy for living donation and HCC (36) | Prospective cohort observational study | 2001 – 2002 | HGF, VEGF | Liver regeneration |
| Effimova 2005 (45) | Hepatectomy for living donation (18) | Not specified | Not specified | HGF, VEGF | Liver injury / regeneration |
| Guidi 2003 (31) | Hepatectomy for various lesion (8) | Prospective cohort observational study | 1997 – 1998 | IL-1, IL-6 | Liver injury / regeneration |
| Justinger 2008 (50) | Hepatectomy for various lesions (20) | Prospective cohort observational study | 2006 – 2007 | HGF, VEGF | Liver injury / regeneration |
| Kimura 1996 (37) | Hepatectomy for various lesions (20) | Prospective cohort observational study | Not specified | IL-6 | Liver injury / regeneration |
| Kimura 2004 (36) | Hepatectomy for various lesions (64) | Not specified | 2001 – 2002 | IL-6 | Liver injury / regeneration |
| Kimura 2006 (38) | Hepatectomy for various lesions (128) | Not specified | 2001 – 2003 | IL-6 | Liver / organ failure |
| Kornasiewicz 2015 (39) | Hepatectomy for various benign lesions (22) | Not specified | Not specified | IL-6, HGF | Liver injury / regeneration |
| Krieg 2006 (46) | Hepatectomy for various malignant lesions (22) | Prospective cohort observational study | Not specified | HGF | Liver injury / regeneration |
| Maeda 1999 (40) | Hepatectomy for various malignant lesions (24) | Not specified | 1995 – 1997 | IL-6 | Liver failure |
| Matsumoto 2013 (47) | Hepatectomy for living donation (16) | Prospective cohort observational study | 2000 – 2010 | HGF | Liver injury / regeneration |
| Mizuguchi 2004 (53) | Hepatectomy for various lesions (37) | Retrospective study | 2002 – 2003 | HGF, HA | Liver failure |
| Sasturkar 2016 (41) | Hepatectomy for living donation (25) | Prospective cohort observational study | 2014 – 2014 | IL-6, HGF | Liver injury / regeneration |
| Sparrelid 2018 (42) | Hepatectomy for metastasis from colorectal cancer (10) | Prospective cohort observational study | 2012 – 2014 | IL-6, HGF, VEGF | Liver injury / regeneration |
| Starlinger 2015 (52) | Hepatectomy for various lesions (157) | Prospective cohort observational study | Not specified | VEGF | Liver failure |
| Takeuchi 1997 (48) | Hepatectomy for various lesions (24) | Not specified | Not specified | HGF | Liver failure |
| Tani 1994 (49) | Hepatectomy for various lesions (21) | Not specified | Not specified | HGF | Liver injury / regeneration |
| Tomiya 1992 (51) | Hepatectomy for various malignant lesions (14) | Not specified | Not specified | HGF | Liver injury / regeneration |
| Yachida 2000 (54) | Hepatectomy for various malignant lesions (36) | Retrospective study | 1992 – 1997 | HA | Liver failure |
| Yachida 2009 (55) | Hepatectomy for various lesions (131) | Retrospective study | 1992 – 2007 | HA | Liver failure |
